# Disrupted functional brain network associated with presence of hallucinations in Parkinson’s disease

**DOI:** 10.1093/braincomms/fcaf185

**Published:** 2025-05-16

**Authors:** Marcella Montagnese, Mitul A Mehta, Dominic ffytche, Michael Firbank, Rachael A Lawson, John-Paul Taylor, Edward T Bullmore, Sarah E Morgan

**Affiliations:** Department of Clinical Neurosciences, University of Cambridge, Cambridge CB2 0SZ, UK; Department of Neuroimaging, Institute of Psychiatry, Psychology & Neuroscience, King’s College London, London SE5 8AF, UK; Department of Neuroimaging, Institute of Psychiatry, Psychology & Neuroscience, King’s College London, London SE5 8AF, UK; Department of Old Age Psychiatry, Institute of Psychiatry, Psychology & Neuroscience, King’s College London, London SE5 8AB, UK; Translational and Clinical Research Institute, Newcastle University, Newcastle Upon Tyne NE1 7RU, UK; Translational and Clinical Research Institute, Newcastle University, Newcastle Upon Tyne NE1 7RU, UK; Translational and Clinical Research Institute, Newcastle University, Newcastle Upon Tyne NE1 7RU, UK; Department of Psychiatry, University of Cambridge, Cambridge CB2 0SZ, UK; Department of Psychiatry, University of Cambridge, Cambridge CB2 0SZ, UK; Department of Computer Science and Technology, University of Cambridge, Cambridge CB3 0FD, UK; School of Biomedical Engineering and Imaging Sciences, King’s College London, London SE1 7EH, UK

**Keywords:** Parkinson’s disease, Parkinson’s psychosis, hallucinations, resting-state fMRI, network neuroscience

## Abstract

Hallucinations negatively impact quality of life in Parkinson's disease, yet their neural mechanisms remain poorly understood, particularly in early disease stages. This study aimed to identify functional connectivity differences associated with visual hallucinations in early Parkinson's disease and to validate these findings across independent datasets. Resting-state functional MRI data from two independent studies were used: the ‘Parkinson's Progression Markers Initiative’ dataset was used as a discovery cohort (*N* = 25 hallucinators, *N* = 56 non-hallucinators) and the ‘Incidence of Cognitive Impairments in Cohorts with Longitudinal Evaluation’ dataset as replication (*N* = 49 hallucinators, *N* = 55 non-hallucinators overall). Group differences in functional connectivity were assessed within predefined cytoarchitectonic cortical classes and functional networks, followed by whole-brain analysis using Network-Based Statistics. This method identified a subnetwork of reduced functional connectivity in hallucinators, connecting regions involved in the default mode, somatomotor and attentional networks. Associations with clinical measures—including hallucination severity, motor symptoms, cognition and attention—were evaluated. Reduced functional connectivity in hallucinators was significantly associated with baseline and future motor symptoms, cognition and attention in the main cohort and with hallucination severity in the independent cohort. The identified functional subnetwork offers a potential direction for future research on Parkinson's disease psychosis.

## Teaser

Montagnese *et al*. report a disrupted functional brain network associated with hallucinations in Parkinson’s disease, including cortical regions implicated in the attentional and default mode networks. Using resting-state functional MRI from two cohorts, they link reduced functional connectivity within the network identified to hallucination severity, cognition and future clinical symptoms.

## Graphical Abstract

**Graphical Abstract:**
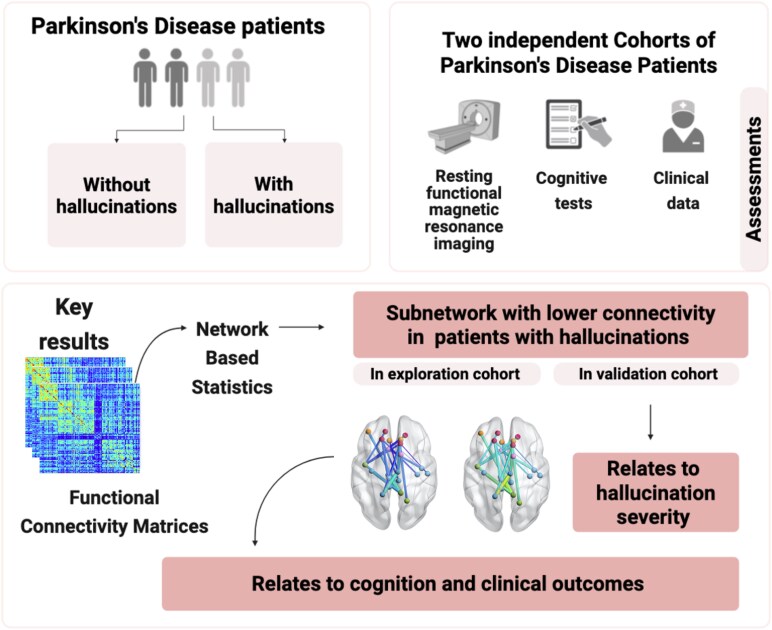

## Introduction

Psychosis is a common yet underappreciated feature of Parkinson's disease (PD), with estimates suggesting half of the 10 million PD patients worldwide experience psychosis at some point during their illness.^1^ Symptoms include hallucinations, predominantly visual, which have been shown to negatively affect patients’ and carers’ quality of life and predict dementia.^2,3^ However, the mechanisms underlying visual hallucinations (VH) and their relationship to cognitive processing and clinical outcomes remain poorly understood. VH might be underpinned not just by regions with neural pathology, but also by unaffected regions within broader functional networks.^4-6^ Converging evidence suggests the contribution of attentional network dysfunctions and an imbalance in top–down and bottom–up perceptual processing,^7^ implicating multiple regions and functional networks.^5,6,8-12^

Against this backdrop, several complementary models have emerged to explain Parkinson's disease psychosis (PDP). While initial explanations focused on dopaminergic medication effects, as reviewed by Pagonabarraga *et al*.^6^ and ffytche *et al.*^2^ understanding has evolved to encompass both circuit-specific and network-level explanations. Two particularly influential frameworks are the Perception–Attention–Deficit (PAD)^13^ and the Attentional Network Dysfunction^14^ model. The PAD model proposes that patients experience deficits in sensory activation and attentional binding. Hallucinations arise from erroneous sensory activation of inappropriate proto-objects (early visual processing where sensory inputs compete for recognition as distinct objects in visual awareness) and failures in attentional binding. The Attentional Network Dysfunction model also attributes VH to attentional control deficits, specifically from perturbations in the interactions among Dorsal Attention Network (DAN), Ventral Attention Network (VAN) and Default Mode Network (DMN)—with experimental work supporting this.^15,16^

Thalamic dysregulation models add that subcortical drivers play a pivotal role: the thalamus, as a sensory relay and regulator of cortical rhythms, can induce network-wide imbalances. Middleton and Strick^17^ provided early evidence that basal ganglia output reaches visual cortical areas via the thalamus, suggesting that dysfunction in this loop could alter visual processing, potentially leading to hallucinations. More recently, thalamocortical dysrhythmia has been proposed as a mechanism for PDP.^18,19^ In this view, PD-related thalamic dysfunction (exacerbated by brainstem cholinergic degeneration^20^) induces abnormal theta-frequency rhythms in cortical circuits. The result is an inhibited frontal attention network and an overactive DMN, effectively ‘decoupling’ internal mentation from external reality.

To date, a large number of functional connectivity (FC) studies have examined network disruptions in PDP (as reviewed by Pagonabarraga *et al*.)^6^—using both task-based and resting-state approaches.^7,9,12,16,21-24^ While these studies have provided important insights into network dysfunction in PDP,^12,25-28^ questions remain about how circuit-level and network-level disruptions might interact to produce hallucinations, with few having performed whole-brain, data-driven analyses.^9,10^ Our study therefore aims to bridge this gap using network-based statistics (NBS), a data-driven network science approach that allows detection of altered sub-networks while controlling for multiple comparisons across the connectome. Our whole-brain NBS analysis enables identification of subtle network-level alterations that may not be captured when examining individual connections or constrained to canonical networks. We additionally examine connectivity within established resting-state networks in order to aid comparisons to previous work.

To address these questions, we formulated the following aims and hypotheses:

- Investigate FC markers in PD patients with hallucinations by:
  - Evaluating group differences in global connectivity. Based on prior research,^9^ we hypothesized reduced global FC in patients compared to healthy controls (HC), with more pronounced reductions in patients were grouped into visual hallucination (PDVH) than non-visual hallucination (PDNOVH) groups.
  - Comparing group differences across Yeo functional networks and von Economo classes. We expected lower FC in attentional networks (DAN and VAN) and higher FC in the DMN in PDVH.
  - Using NBS to identify brain-wide connectivity differences associated with VH in PD.
- Assess whether any significant results from A) replicated in an independent cohort.
- Explore the relationships between hallucination-specific network differences and clinical/cognitive variables, both cross-sectionally and longitudinally.

## Materials and methods

### Primary cohort - Parkinson’s Progression Marker Initiative

Our primary sample came from the Parkinson's Progression Marker Initiative (PPMI) and included resting-state fMRI data from PD patients and age-matched HC. Detailed exclusion criteria for the PPMI cohort are given in Supplementary Section 4. PDVH or PDNOVH groups based on scoring ≥1 on Question 1.2 of the Movement Disorder Society Unified Parkinson's Disease Rating Scale (MDS-UPDRS^29^)—‘*Over the past week have you seen, heard, smelled or felt things that were not really there?*’—at one or more concomitant/previous assessments (*N* = 12 scored 1, *N* = 11 scored 2 and *N* = 2 scored 3). The overall sample included: *N* = 25 PDVH patients, *N* = 56 PDNOVH patients and *N* = 24 HC.^30^ By design, most PPMI patients were at early stages of their disease.

#### Cognitive measures

Global cognition was assessed with the Montreal Cognitive Assessment (MoCA)^31^; visuospatial function with the Benton Judgement of Line Orientation task^32^; and executive function with semantic fluency (total number of animals named in 1 min).^33^ Attention was measured with the letter–number sequencing task,^34^ and episodic memory with the Hopkins Verbal Learning Test (VLT) Total Delayed Recall^35^ (sum of Recall Trial 1–3).

#### Clinical measures

Motor symptom severity was measured using the motor subscale of total MDS-UPDRS-III.^29^ PD duration was recorded as number of years since PD diagnosis. Levodopa-equivalent daily dose (LEDD) was the daily dose (milligram) on assessment day (See PPMI manual^36^). Sleep disorders were assessed with the rapid eye movement (REM) Sleep Behaviour Disorder Screening Questionnaire (RBDSQ).^37^ CSF sampling and analysis are described in detail in the PPMI manual.^36^ The following were available for a subset of patients: CSF β-amyloid (Aβ1–42 pg/mL), tau proteins total (T-Tau) and α-synuclein concentration levels.

### Secondary cohort - ICICLE-PD

PPMI was designated as the discovery cohort, with the Incidence of Cognitive Impairment in Cohorts with Longitudinal Evaluation-PD (ICICLE-PD) study^38^ serving as the replication cohort due to the sequence in which the datasets were received. Inclusion and exclusion criteria for ICICLE-PD are given in Supplementary Section 4. A subset of participants completed the North East Visual Hallucination Interview (NEVHI),^30^ a semi-structured interview developed by Mosimann *et al.*^39^ covering the phenomenology of VH. Patient and caregiver versions were used. A positive score (‘Yes’) on one or more of NEVHI Part A screening questions 1.1–1.6 about presence of hallucinatory experiences (such as Question 1.1 ‘Do you feel like your eyes ever play tricks on you? Have you ever seen something (or things) that other people could not see?’) was used to categorize patients as PDVH or PDNOVH (see Supplementary Section 5). To systematically evaluate FC differences, we first analysed the full ICICLE-PD cohort (*N* = 104).

#### Clinical and cognitive measures

Hallucinations severity was computed using the NEVHI by following the guidelines outlined in its manual.^39^ This involved multiplying scores of hallucination frequency, hallucination duration and severity factors specific for the type of hallucination (see Supplementary Section 6). As for the PPMI cohort, global cognition was assessed with the MoCA^31^ and motor symptom severity was measured using the motor subscale of total MDS-UPDRS-III.^29^

### Statistical analysis of demographics

Demographic and clinical characteristics of PDVH, PDNOVH and HC groups were compared using one-way ANOVA for normally distributed and Kruskal–Wallis for non-normally distributed variables, with χ^2^ for categorical variables. For variables only relevant to patients, independent *t*-tests and Mann–Whitney tests were used. Shapiro–Wilk tests were used to assess normality.

### Data pre-processing

Full details on the acquisition of both Magnetization Prepared Rapid Acquisition Gradient Echo (MPRAGE) T1-weighted sequence and resting-state fMRI data in PPMI and ICICLE-PD can be found in Supplementary Section 1. rsfMRI data for both cohorts were pre-processed according to a published pipeline^40^ described in Supplementary Section 2. Denoising of motion artefacts was done with BrainWavelet toolbox's wavelet despiking.^40^ Brain surfaces were parcellated using an atlas with 308 cortical regions of approximately equal size based on a subdivision of the Desikan–Killiany atlas.^41^ FC matrices were calculated using Pearson's correlation of pairwise normalized wavelet coefficients between regions.

Participants with mean framewise displacement (FD) > 0.6 mm were excluded to reduce motion artefacts^42^: *N* = 4 PDVH, *N* = 8 PDNOVH and *N* = 1 HC were excluded in PPMI; and *N* = 3 PDNOVH and *N* = 5 PDVH in ICICLE-PD. Mean FD was regressed from each FC matrix edge to remove motion correlations and distance-dependent motion effects (Supplementary Section 3). In ICICLE-PD, further 9 patients (4 PDVH and 5 PDNOVH) were excluded due to failed co-registration, and 1 PDNOVH was excluded for having many drop-out regions. (For further details on the specific software used for pre-processing, see Supplementary Section 11.)

### FC analyses across Yeo networks and von Economo classes in PPMI

We first calculated group differences in mean FC averaged across the whole brain and averaged within each von Economo cytoarchitectonic class and Yeo network. Differences were calculated between the three groups using linear models (including age, sex and age*sex as covariates). To assess sensitivity, analyses were repeated including MoCA, UPDRS-III and LEDD as covariates. After removing outliers (±2 SDs), sample sizes were: PDVH *N* = 22, PDNOVH *N* = 55, HC *N* = 22.

### NBS in PPMI

We used NBS^43^ to identify a subnetwork of edges that differed between the PDVH and PDNOVH groups, co-varying for sex and age. NBS analyses were run with 5000 permutations to ascribe a family-wise error rate (FWER)-corrected *P*-value (<0.05). All analyses were performed using the NBS Toolbox v1.2.^43^ Additional sensitivity analyses were run to confirm that the average NBS connectivity was not correlated with mean FD. Correlations were run between average NBS FC and average FD in each group (see Supplementary Section 3.1).

### NBS replication analysis in ICICLE-PD cohort

We tested whether the NBS subnetwork identified using the PPMI cohort replicated in the ICICLE-PD cohort. Replication was assessed using two approaches: (i) Calculating total FC within the NBS network for each individual in the ICICLE-PD cohort and applying a linear model to test group differences; and (ii) using the NBS toolbox to directly compare PDVH and PDNOVH group differences in ICICLE-PD, using the same t-threshold as in PPMI. For the whole ICICLE-PD cohort we covaried for age, sex, UPDRS-III, MoCA scores, LEDD and education.

### Relating NBS connectivity to clinical and cognitive variables

#### Primary PPMI cohort

To understand the relationship between the NBS network defined in PPMI and clinical and cognitive variables of interest we ran a principal component analysis (PCA) on all clinical and cognitive variables available with R's ‘factoextra’ and ‘factominer’ (‘v2.4’). We then related the PCA components to average NBS subnetwork connectivity with Spearman's correlations—run separately for PDVH and PDNOVH groups.

Further exploratory Spearman correlation analyses were conducted to assess whether average NBS connectivity was associated with cognitive decline and clinical outcomes at baseline and at follow-up. To maximize data availability, a follow-up period of 4 years (±1 year) was used. The variables included in the analysis were attentional performance, MoCA, REM sleep behavior disorder (RBD) and MDS-UPDRS-III scores.

#### Secondary ICICLE-PD cohort

In the PDVH group from ICICLE-PD, we related average FC within the masked PPMI NBS network to hallucination severity scores from the NEVHI. A linear regression was run with masked NBS FC as the independent variable and severity as the dependent variable, with a sensitivity analysis controlling for disease severity (MDS-UPDRS-III).

## Results

### Primary sample (PPMI) characteristics

There were no statistically significant group differences across shared demographic and cognitive variables, nor in medication and motor symptoms severity between PDVH and PDNOVH (see Table 1).

**Table 1:** Summary demographics for the primary and secondary cohorts, including the group of matched ICICLE-PD patients included in the NBS replication analysis

	Primary cohort - PPMI							ICICLE-PD Full Cohort				
Variable	HC *N* = 24		PDNOVH *N* = 56		PDVH *N* = 25			PDNOVH *N* = 55		PDVH *N* = 49		
	Mean	SD	Mean	SD	Mean	SD	*P*-val	Mean	SD	Mean	SD	*P*-val
Age	61.67	10.58	62.74	10.0	61.48	9.36	0.88	65.2	11.3	65.7	9.89	0.65
Gender (F/M)	4/20		14/42		9/16		0.16	23/32		11/38		0.03*
MDS-UPDRS-III			20.64	9.94	25.88	11.16	0.08	23.9	11.4	29.1	10.5	0.01*
Hoehn & Yahr			1.77	0.47	1.72	0.46	0.69	1.89	0.59	2.02	0.69	0.32
PD duration (years)			2.95	1.74	3.6	1.69	0.15	0.46	0.39	0.55	0.42	0.22
LEDD (mg/day)			434.6	252.44	463.94	262.49	0.53	164.3	99.4	188	170	0.68
Education (years)	16.00	3.07	15.8	2.45	14.44	2.97	0.07	13.71	4.05	12.27	3.78	0.07
MoCA total	27.75	1.62	27.55	2.11	25.96	4.29	0.43	26.2	3.16	24.91	3.46	0.03*
JLO total	13.43	1.41	13.3	1.65	12.52	2.31	0.30					
MoCA visuoconstruction	3.50	0.58	3.54	0.79	3.28	1.14	0.66					
Hopkins VLT	46.41	10.19	48.80	9.22	46.36	16.2	0.56					
Letter-Num-Seq	11.17	1.89	10.96	2.80	9.68	3.55	0.40					
Fluency (animals)	21.00	4.79	22.64	6.00	20.96	5.51	0.32					
CSF *α*-syn^a^			1400	618	1294	446	0.62					
CSF β-amyloid^a^			852	353	825	331	0.85					
CSF T-Tau^a^			163	52.9	156	50.7	0.69					
Hall. Severity^b^										4.05	3.61	

Significant *P-values <0.05.

MoCA, Montreal cognitive assessment; JLO, judgement of line orientation; CSF, cerebrospinal fluid; LEDD, Levodopa-equivalent daily dose; MDS-UPDRS, Movement Disorders Unified Parkinson's Disease Rating Scale; VLT, verbal learning test.

^a^Samples for the CSF measures were PDVH *N* = 23; PDNOVH *N* = 49 due to data missingness. ^b^Sample for ICICLE PDVH Hallucination Severity was *N* = 34 due to data missingness.

### Replication sample (ICICLE-PD) characteristics

In the full ICICLE-PD cohort (*N* = 104), the PDVH and PDNOVH groups were unmatched on key demographics and clinical variables (sex, disease severity, MoCA—see Table 1). Supplementary analyses with Sample 2, where ICICLE-PD patients were matched both with each other and with PPMI can be found in Supplementary Table 1, and Supplementary Sections 4 and 9.

### Group differences in FC in PPMI cohort

**Figure 1:**
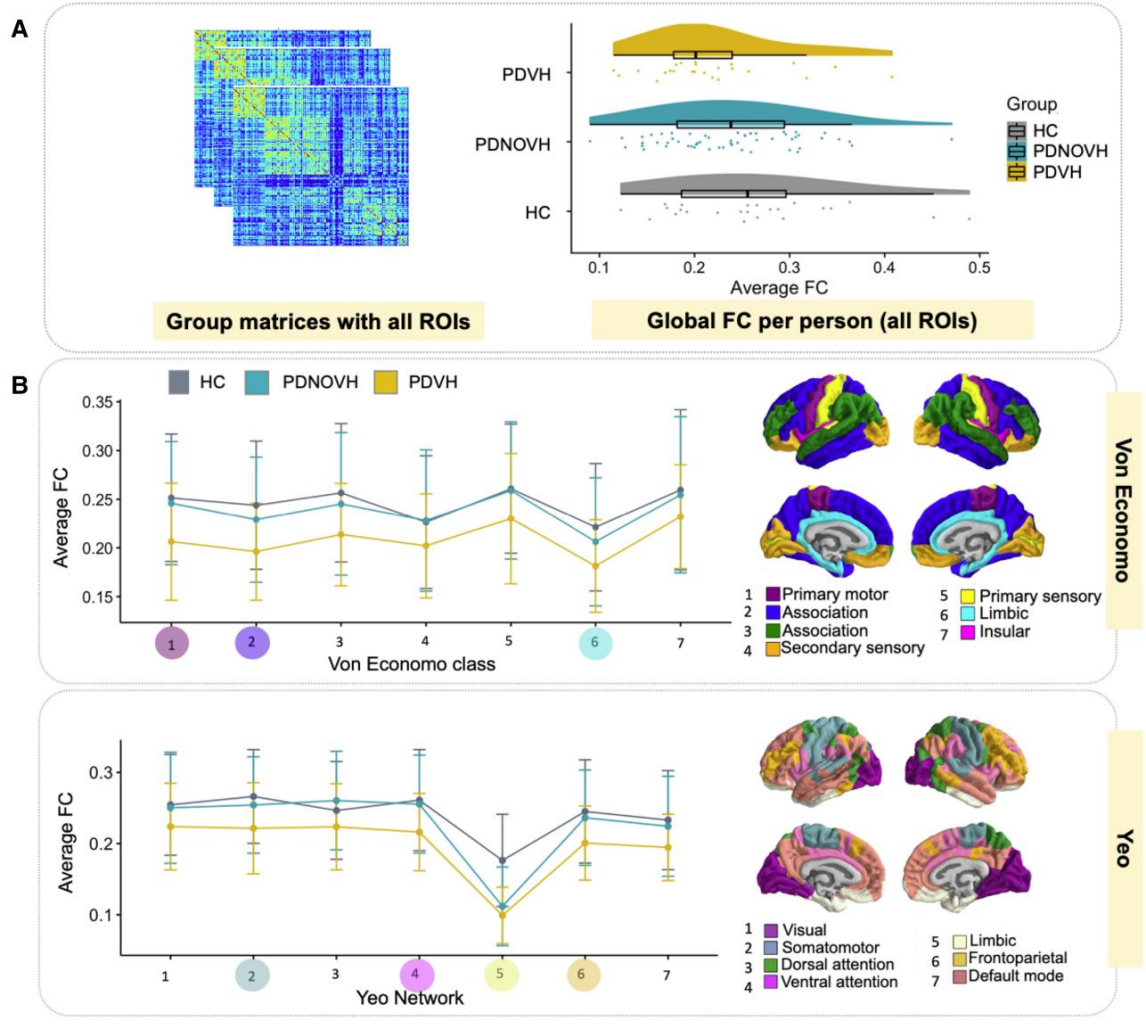
**Group differences in FC at the global level and within previously defined von Economo and Yeo functional networks.** (**A**) Global FC differences between groups. *Left*: average FC matrix per group, for illustrative purposes only. *Right*: differences in global FC (median ± inter-quartile range) across the three groups (Kruskal–Wallis test, test-statistic = 2.65, *P* = 0.266; PDVH *N* = 22, PD NOVH *N* = 55, HC *N* = 22). (**B**) Average FC within von Economo cytoarchitectonic classes and Yeo canonical functional networks. Error bars indicate SD. Coloured dots at the *bottom* of the *x*-axis indicate networks/classes with significant group effects (*P* < 0.05) ‘before’ FDR correction. Group comparisons performed using linear models with age, sex and age × sex as covariates (*N* = 99 total: PDVH = 22, PD NOVH = 55, HC = 22). None of these group differences was statistically significant after FDR correction, with the exception of the limbic network when comparing patients with HC. PD, Parkinson's disease; HC, healthy controls; VH, visual hallucinations; NOVH, no visual hallucinations.

In the PPMI cohort, FC decreased across groups with highest values in HC and lowest values in PDVH (see Fig. 1A), however, differences were not statistically significant (‘*Kruskal–Wallis test-statistic*’ = 2.65, *P* = 0.266).

Group comparisons of FC across functional Yeo networks and von Economo cytoarchitectonic classes (Fig. 1B) showed that after false discovery rate (FDR) correction, only differences in the functional Yeo limbic network remained significant (*p*_FDR_ = 0.007), with both PD groups differing from HC. Initial uncorrected analyses suggested broader differences across several networks and classes, but these did not survive multiple comparison correction (full statistical details in Supplementary Table 2—Supplementary Section 7).

### NBS subnetwork related to hallucinations in PPMI cohort

**Figure 2:**
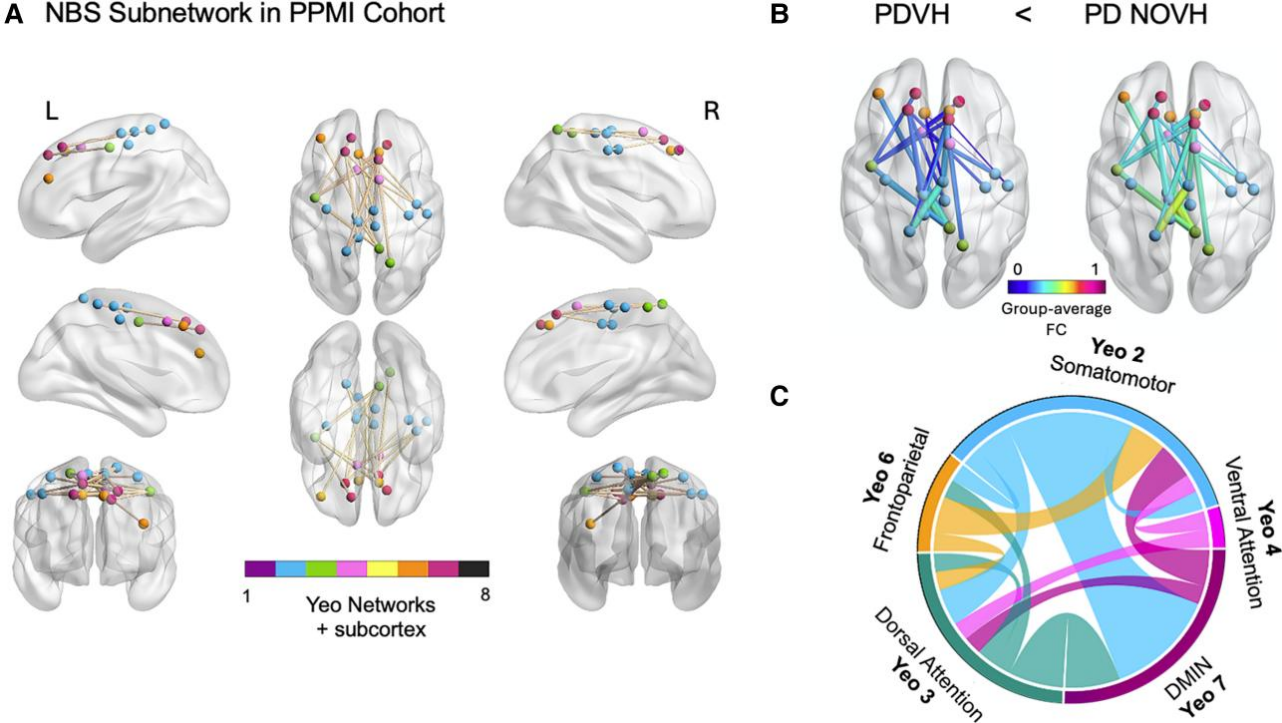
**NBS differences in FC between the VH and NOVH groups, in the PPMI cohort.** Nodes are colour-coded consistently across figures according to their mapping to the 7 Yeo canonical functional networks. This node colour scheme is deliberately consistent throughout to maintain network correspondence, as generated by the BrainNet Viewer Toolbox. (**A**) Network of edges showing reduced FC in the VH group (*N* = 25) compared to the NOVH group (*N* = 56), identified by NBS analysis with *t*-threshold = 3.8, *P* = 0.04, 5000 permutations. Here, the edge colour does not represent a statistical or connectivity scale as it was chosen simply for visibility in the 3D rendering. (**B**) The same NBS-derived set of edges are shown, but now coloured to reflect group-averaged FC values (low to high). The node colours remain the same as in **A** to maintain network correspondence. (**C**) Connectogram visualization highlighting the most salient Yeo networks. PD, Parkinson's disease; VH, visual hallucinations; NOVH, no visual hallucinations.

NBS analyses revealed a statistically significant network of reduced FC in PDVH compared to PDNOVH (Fig. 2) comprising 22 nodes and 23 edges (t-threshold = 3.8, *P* = 0.04, 5000 permutations). Connections were mainly between regions located in the DMN, somatomotor, DAN and VAN networks (see Fig. 2C and Supplementary Table 3). The edge-pairs with the highest *t*-statistics were between the DMN and DAN, DMN and somatomotor, and DAN and somatomotor networks. No significant NBS network was found in the opposite direction (PDVH FC > PDNOVH).

Additionally, we conducted an exploratory follow-up analysis to examine whether regions showing connectivity alterations in the NBS subnetwork also experience greater structural differences, as indexed by regional grey matter (GM) volume. We computed Cohen's d effect sizes for group differences (VH versus NOVH) in GM volume across all brain regions and compared the magnitude of these differences between NBS and non-NBS regions. The analysis revealed no significant difference in effect sizes between NBS regions (mean absolute effect size = 0.202) and non-NBS regions (mean absolute effect size = 0.249; *t* = −1.184, *P* = 0.237) (See Supplementary Section 8).

### Replication of NBS subnetwork in ICICLE-PD cohort

We assessed whether the PPMI NBS subnetwork replicated in an independent dataset. For the ICICLE-PD full cohort (*N* = 104) we included the following covariates: age, sex, UPDRS-III, MoCA scores, LEDD and education. With Method 1, there was a difference in masked FC within the PPMI-defined NBS network between the PDVH and PDNOVH groups (*β* = −0.11, *P* < 0.05, with overall model *R*^2^ = 0.16, adjusted *R*^2^ = 0.09, *F*(7,96) = 2.54, *P* < 0.05), hence FC within the PPMI-defined NBS network was significantly reduced in the PDVH group. When using NBS to directly calculate PDVH versus PDNOVH connectivity differences in the same ICICLE-PD dataset we found no significant NBS network (‘*threshold*’ = 3.8, contrast PDVH < PDNOVH, including all of the same covariates).

We also conducted a *post hoc* NBS analysis in a smaller subset (Sample 2) where PDVH and PDNOVH groups were matched to each other and to PPMI on key variables. This revealed a significant reduction in connectivity within the PPMI NBS subnetwork in PDVH compared to PDNOVH, as well as a significant NBS-derived network in the ICICLE sub-sample (refer to Supplementary Table 4 and Supplementary Fig. 4 in Supplementary Section 9).

**Figure 3:**
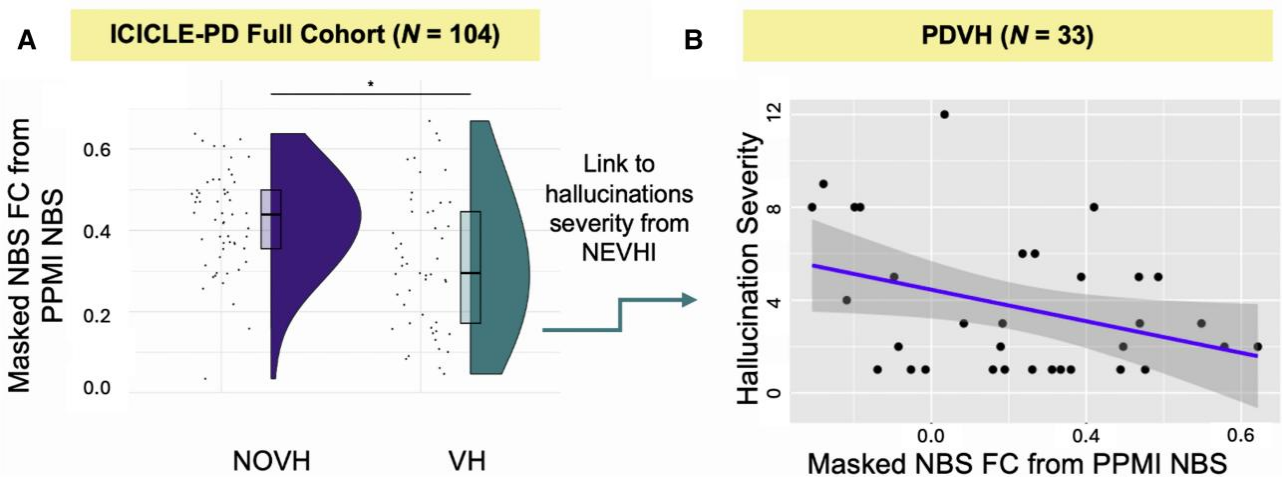
**NBS network replicated in the ICICLE-PD dataset.** (**A**) Raincloud plot of FC within PPMI-defined NBS subnetwork in ICICLE-PD cohort (*N* = 104); boxes show quartiles, data points indicate individual values (*t*-test *β* = −0.11, *P* < 0.05). (**B**) Visualization of the relationship between hallucination severity score and FC within NBS subnetwork in PDVH group from ICICLE-PD (assessed with a linear model, *N* = 33, *β* = −6.78, *P* = 0.04; *R*^2^ = 0.12, adjusted *R*^2^ = 0.10, overall model *P* = 0.04), each data point representing an individual participant. PD, Parkinson's disease; VH, visual hallucinations; NOVH, no visual hallucinations. *Indicates significant values (*P* < 0.05).

We then related average FC within the PPMI NBS network (see Fig. 3A) to hallucination severity scores from the NEVHI. In the full ICICLE-PD Cohort, sample size for PDVH was *N* = 33 due to data missingness and after removal of *N* = 1 outlier (defined as values falling outside 1.5 times the interquartile range). A linear regression was performed with masked NBS FC as the independent variable and severity as the dependent variable, with a sensitivity analysis controlling for disease severity (MDS-UPDRS-III). Results showed that FC within the PPMI-defined NBS subnetwork was significantly associated with hallucination severity in the PDVH group from ICICLE-PD full cohort (*β* = −6.78, *P* = 0.04; *R*^2^ = 0.12, adjusted *R*^2^ = 0.10, overall model *P* = 0.04; Fig. 3B). After controlling for disease severity (MDS-UPDRS-III), masked NBS FC remained a significant individual contributor (*β* = −6.89, *P* = 0.04), although the overall model was not significant (*R*^2^ = 0.15, adjusted *R*^2^ = 0.09, *P* = 0.08).

### Relation between NBS connectivity and clinical/cognitive measures (PPMI cohort)

**Figure 4:**
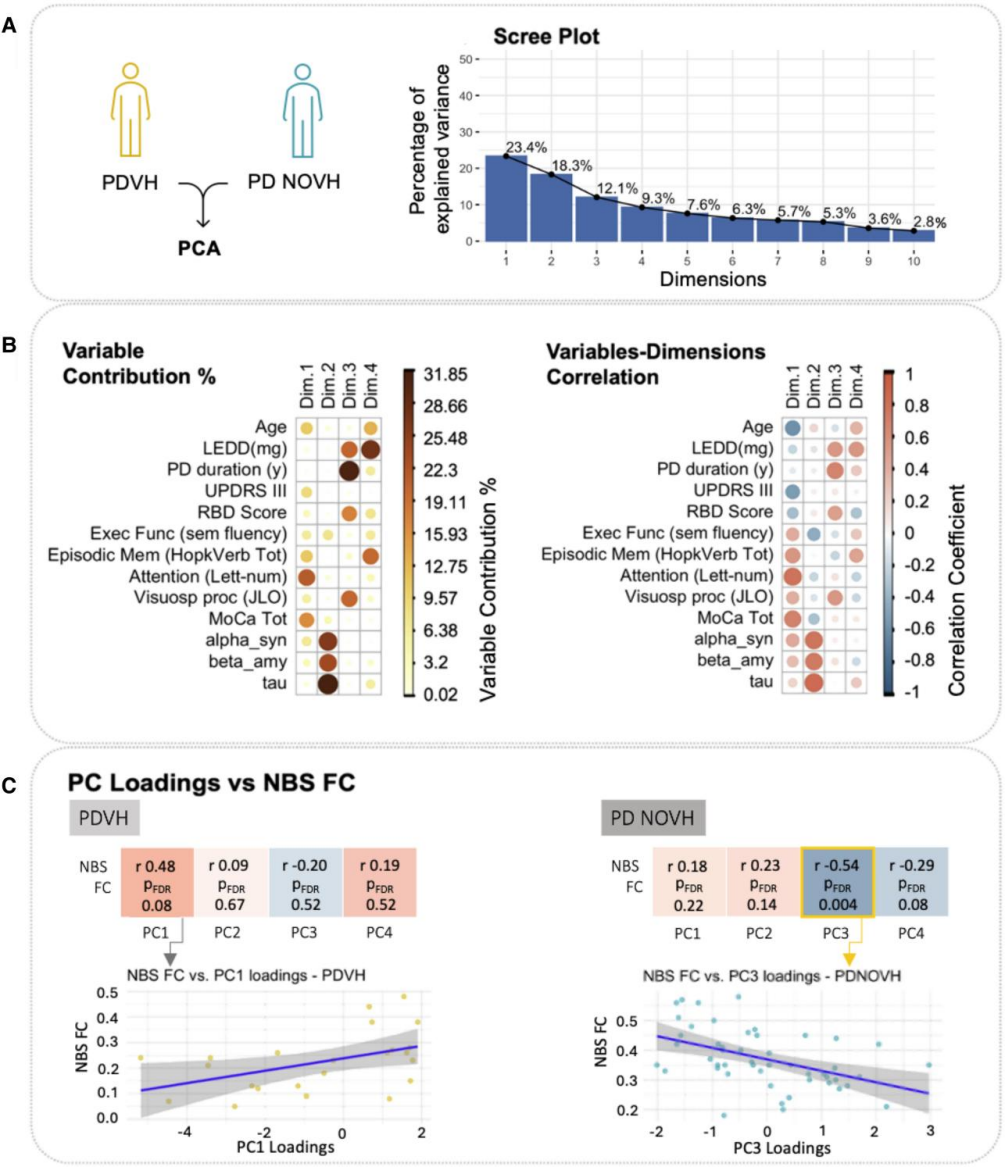
**PCA of clinical and cognitive variables, for the PPMI dataset.** (**A**) Scree Plot showing the amount of variance explained by the PCs. Only components with eigenvalues >1 were selected, resulting in four PCs. *N* = 69, including *N* = 23 PDVH, *N* = 46 PDNOVH after excluding cases with missing CSF data. (**B**) On the left, descriptive visual representation of the first 4D with a plot of the relative contribution of each variable (in percent, %) to each of the dimensions (*N* = 23 PDVH, *N* = 46 PDNOVH). On the *right*, descriptive visual representation of the Spearman correlations between each variable and each dimension (*N* = 23 PDVH, *N* = 46 PDNOVH). (**C**) Spearman's correlation analyses linking NBS functional connectivity (NBS FC) to each of the four PCs for each group separately. On the *bottom* left, we show a correlation plot in the PDVH group for NBS FC versus the loading of the PC1 dimension; on the *bottom* right we show a correlation plot in the PDNOVH group for NBS FC versus the loading of the statistically significant PC3 dimension. In both of these correlation plots, each data point represents a patient (PDVH on the *left* and PDNOVH on the *right*). FDR values shown represent *P*-values after FDR correction. PD, Parkinson's disease; VH, visual hallucinations; NOVH, no visual hallucinations.

We related FC within our NBS subnetwork to clinical and cognitive measures from the PPMI cohort (*N* = 69, including *N* = 23 PDVH, *N* = 46 PDNOVH, after excluding cases with missing CSF data). Principal component analysis (PCA) identified 4D with eigenvalues >1, explaining 63.03% of total variance. High PC1 was associated with lower age and better attention, executive function and general cognition (MoCA), and to a lesser extent, lower motor severity. High PC2 was related to higher CSF levels (alpha-synuclein, t-Tau and beta-amyloid). High PC3 was related to longer PD duration, higher LEDD, worse RBD symptoms and better visuospatial processing. High PC4 was related to higher LEDD, better memory and older age (see Fig. 4).

In PDVH, connectivity in the NBS subnetwork positively correlated with PC1, but the relationship was not significant after FDR correction (*ρ* = 0.48, *P*-val = 0.02, *P*-val_FDR_ = 0.08).

In PDNOVH, NBS FC was negatively correlated with PC3 both before and after FDR correction (*ρ* = −0.54, *P*-val = 0.001, *P*-val_FDR_ = 0.004), indicating that lower NBS FC was associated with higher LEDD, longer disease duration, worse RBD symptoms and better visuospatial processing.

### Relation between NBS connectivity and future clinical outcomes in PPMI cohort

**Figure 5:**
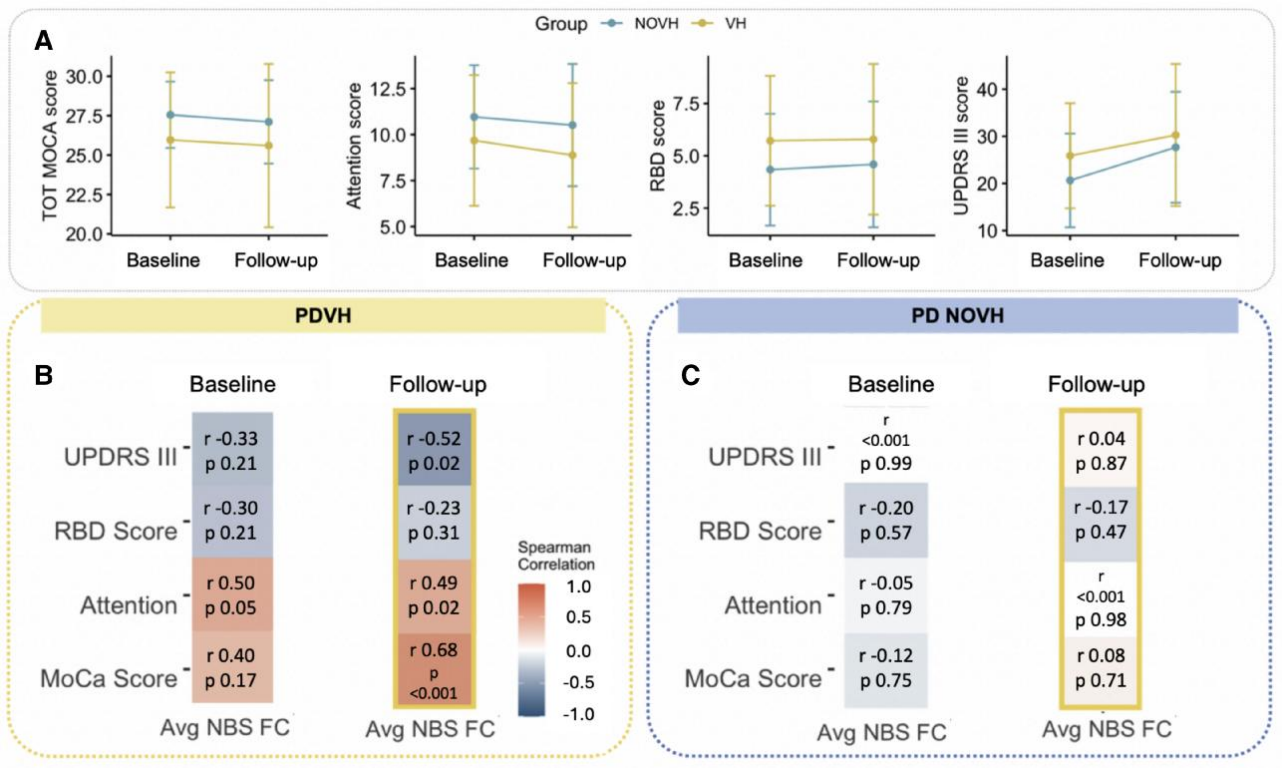
**Relation between NBS connectivity and future clinical outcomes.** (**A**) Plot of mean and SD for each variable per group at baseline and at follow-up time of 4 years (±1 year); PDVH *N* = 25; PD NOVH *N* = 56. (**B**) Spearman correlations between clinical measures and FC within the NBS subnetwork at baseline and at follow-up for the PDVH group (*N* = 25). Spearman correlation coefficients (*ρ*) are given, alongside *P*-values after FDR correction. (**C**) The same correlations for the PDNOVH group (*N* = 56). For individual data points shown, each dot represents a single participant's score for that variable. Correlation coefficients (*ρ*) and FDR-corrected *P*-values are reported. PD, Parkinson's disease; VH, visual hallucinations; NOVH, no visual hallucinations.

Lastly, we examined the association between baseline NBS connectivity and future clinical outcomes (see Fig. 5 and Supplementary Table 5 in Supplementary Section 10). In the PDVH group, average NBS FC was positively correlated with better attention at both baseline and follow-up, and better cognition (MoCA total score) at follow-up. Lower NBS FC at baseline were related to worse (higher) MDS-UPDRS-III scores at follow-up. No significant correlations were found for the PDNOVH group.

## Discussion

NBS identified a network of reduced FC associated with VH in PD patients. The NBS network included connections within and across the dorsal attention, ventral attention and default mode networks. Lower FC within the network was associated with increased hallucination severity, and with baseline and future scores of motor symptom severity, cognition and attention in PD patients with hallucinations.

### Relation to prior models of PDP

The involvement of the DAN, VAN and DMN in the NBS network of reduced FC in PDP was in-line with prior work.^6,8,9,16,26,44,45^ Several network edges connected the DAN with the DMN, the somatomotor network and the frontoparietal network, in-keeping with the central role of the DAN in PDP^10,26,44^ and consistent with both the atentional deficit^14^ and the PAD^13^ models. Further underscoring the notion of attentional impairments in PD hallucinators, many of the NBS-identified nodes map onto anatomical landmarks within the superior frontal gyrus—a principal component of the DAN and a structure implicated in PDP.^8,9^

In particular, our findings are in-line with the work of Hepp *et al.*,^9^ who reported reduced whole-brain connectivity in PD hallucinators, with specific reductions in frontal, occipital and temporal regions. Our findings support this broad hypoconnectivity pattern, but instead of focusing on whole-brain FC metrics, we identified a statistically significant functional subnetwork disrupted in PDVH. This methodological distinction highlights that in our cohort hallucinations were associated with dysfunction within an integrated subnetwork spanning attention, sensory and default mode regions, rather than generalized FC reductions across the whole brain. Tan *et al*.^10^ described a ‘compression’ of the functional hierarchy between unimodal (sensory) and heteromodal (association) networks in hallucinating PD patients, suggesting that higher-order regions exert excessive top–down influence on sensory processing. Our network-level findings may speculatively reflect a complementary aspect of this: while Tan found excessive influence of higher-order regions on sensory processing, we observed reduced connectivity in the normal pathways that maintain distinct functional compartments (attention networks filtering sensory input). This reduced connectivity could relate to weakened functional differentiation between perceptual and cognitive systems.

### Thalamic pathways and downstream cortical alterations

Although our analysis focused only on cortical networks, they should also be considered within the broader context of subcortical influences—particularly thalamic and basal ganglia pathways—which are increasingly recognized as key modulators of large-scale cortical network balance in PD hallucinations.^6,42^ The basal ganglia–thalamic–cortical circuit plays a fundamental role in visual processing and attentional control, with Middleton and Strick^17^ demonstrating that the substantia nigra pars reticulata (SNpr) projects to the inferotemporal cortex via the thalamus, thus providing an anatomical link between subcortical dysfunction and altered visual perception.^17^ This suggests that disruptions in basal ganglia output could directly impair thalamic sensory gating, weakening attentional control over visual processing. Our results align with this model, as DAN–DMN alterations in PDVH may reflect a failure in thalamic regulation of top–down attentional suppression rather than an intrinsic overactivation of the DMN.

Given that our cohort had relatively preserved cognitive function, our results are consistent with the view that thalamic dysregulation might precede widespread cortical atrophy and contributes to network instability in PDVH. This is in-line with work from Zarkali *et al.*,^46^ who observed longitudinal atrophy and white matter loss in the mediodorsal thalamic nucleus in hallucinators, even when cortical atrophy was mild. This mediodorsal nucleus is highly connected with prefrontal and limbic areas, with its degeneration and reduced connectivity likely disrupting the flow of information to frontal executive regions.^20^ As a consequence, this thalamic disconnection could impair attentional gating. Our finding of weakened frontoparietal connectivity in PDVH might be a downstream effect of this: if thalamic input to the prefrontal cortex is compromised, the frontal nodes cannot properly sustain attention or communication with parietal and visual areas, compromising one's ability to regulate cortical network dynamics.

Our results also suggest a complex and nuanced role of the DMN in PD hallucinations. Contrary to one of our hypotheses (ii) and to previous work,^16,28,47^ our results did not reveal hyperconnectivity within the DMN in PDVH, nor between the DMN and other networks. A partial explanation for this discrepancy is that, to our knowledge, no previous studies had examined the DMN with the same whole-brain analytic approach that we employed. Another possible explanation is that studies reporting hyperactivity of the DMN often included older patients with longer disease duration: e.g. in Shine *et al*.^16^ PDVH patients had mean age of 69.3 years and disease duration of 6 years (compared to 61.4 and 3.6 years, respectively in the PPMI cohort). Similarly in Yao *et al.*^28^ the mean age of PDVH patients was 67.6 years old, with an average 10 years disease duration. Furthermore, this pattern of results is particularly relevant in the context of the thalamocortical dysrhythmia proposed by Onofrj *et al.*^18,19^ in previous work, which suggests that thalamic dysfunction results in an ‘inhibited’ frontal attentional network and a ‘decoupled’ DMN, allowing internally generated imagery to intrude on perception. While our lack of findings about DMN hyperconnectivity does not align fully with the model, they could indicate that hallucinations in PD may not just arise from DMN overactivity but also from a selective disruption of attentional control over internally generated percepts. By acknowledging that our results are best interpreted as part of a larger thalamo-cortical system breakdown, it is possible that the hallucination network detected by NBS could partly be a downstream reflection of deeper circuit aberrations.

### Structural connectivity and network topology considerations

Our study focused on ‘FC’ alterations, but it is crucial to also interpret these findings by looking at the relevant literature on structural network disruptions linked to hallucinations in PD. Increasing evidence suggests that white matter connectivity loss in key brain networks may precede and contribute to the functional network breakdown observed in PDVH, reinforcing the idea that hallucinations are driven by both anatomical disconnection and functional dysregulation. Hall *et al*.^48^ used diffusion MRI and graph theory to demonstrate that hallucination severity in PD correlates with widespread white matter disconnection, interpreted as evidence that inefficient information transfer across the brain's modules may give rise to hallucinations by failing to reconcile sensory inputs with higher-order predictions. Similarly, Zarkali *et al*.^49,50^ found that PDVH is associated with the selective loss of high-influence hub regions, leading to diminished network controllability, and noted posterior white matter tract degeneration. These patterns of results align with our findings, as many of the structurally vulnerable hubs identified in these studies overlap with our NBS subnetwork, and we also found disrupted occipital–frontal FC. Such convergence between structural and functional disruptions underscores the interdependence of network integrity at multiple levels, where anatomical vulnerability may create a substrate for functional dysregulation.

### Cognitive significance

In our main cohort, the relationships between NBS network connectivity and clinical/cognitive measures aligned with attentional deficits in PDP, corroborating prior meta-analytic findings of attention, executive function and general cognition being most impaired in hallucinators.^51^ Finally, higher NBS connectivity at baseline correlated both with better performance on attentional tasks and overall cognition, and with worsening motor symptoms severity at follow-up. The specificity of these predictions to the PDVH group suggests again that the NBS network is characteristic of PDP.

### Validation and relation to hallucination severity

A similar pattern of connectivity differences was found in the independent ICICLE-PD cohort. When considering the mean connectivity of the overlapping regions in common with the PPMI subnetwork in the full ICICLE-PD cohort, we observed significantly lower masked mean connectivity in PDVH compared to PDNOVH, after controlling for multiple clinical and demographic variables. However, when directly applying the NBS method in the same dataset, we did not identify a statistically significant subnetwork. The broader clinical and demographic variability in the full ICICLE-PD cohort may have contributed to increased heterogeneity in ‘edge-level’ connectivity patterns, reducing the power to detect significant edge-specific network differences while still allowing detection of aggregated average connectivity differences across the previously identified regions. Prior research suggests that network-level disruptions in PD hallucinations become more pronounced in patients with specific disease profiles,^2,14^ and pooling patients across a broader disease spectrum may have obscured such effects, consistent with the hypothesis that differences in cognitive performance introduce heterogeneity into the network characteristics of different patient groups.^51^ In-line with this, Knolle *et al*.^52^ found that while PD patients with psychosis exhibited significantly higher GM network variability than non-hallucinators, these hallucination-specific alterations were overshadowed by the wider spectrum of PD-related structural changes, resulting in poor classification accuracy at the group level. These findings reinforce that PD pathology may dominate over subtle hallucination-linked disruptions.

In the PDVH group of the ICICLE-PD cohort, more severe hallucinations were related to reduced mean masked NBS FC, establishing an important link between NBS network connectivity and degree of hallucinations. We note that higher severity composite score could mean that lower NBS FC is linked to patients experiencing multiple types of hallucinations or the same symptoms occurring more frequently or lasting longer in time. More work is required to delineate these options.^53^ Unfortunately, with our sample size, we could not stratify by severity or type of hallucination to explore this.

### Limitations and future directions

Some limitations should be noted: (i) participants were not recruited to investigate psychotic symptoms, resulting in a lack of detailed phenotyping about hallucination characteristics. Consequently, reliance on MDS-UPDRS question 1.2 may have included patients with minor and/or complex hallucinations, including experiences beyond the visual domain. Future studies should incorporate more comprehensive measures across sensory modalities^54^; (ii) visual testing was not conducted to assess potential low-level visual deficits,^55^ and hallucination states could not be directly measured during scanning, limiting the ability to examine connections with active hallucinatory episodes versus only hallucination traits; (iii) we lacked comprehensive data on non-anti-Parkinsonian drugs that could induce hallucinations (e.g. anticholinergics and opioids). While LEDD was included as a covariate, other medications may have influenced the results; (iv) sample sizes, whilst comparable to other recent studies, were relatively small; (v) our analysis was confined to cortical regions and future studies should extend these analyses to include subcortical regions given that structures such as the thalamus and basal ganglia may also play a significant role in hallucinations and may drive or modulate the cortical network changes we observed; finally, (vi) while our pre-processing pipeline—including wavelet despiking, regression of mean FD and distance-dependent motion correction—addresses key motion-related confounds and enables a trade-off between data quality and sample retention, we acknowledge that residual motion-related effects, such as spin-history artefacts, may remain. Future studies with larger sample sizes would benefit from applying a more stringent FD threshold (e.g. <0.3 mm) to further minimize motion-related biases while maintaining statistical power.

Future studies could implement alternative methods and frameworks for data-driven analyses and replication, such as template-based-rotation,^56^ alongside combining multimodal imaging (such as diffusion MRI) with NBS analyses of functional data to help better clarify how structural disconnections might contribute to sub-networks dysregulation in PDVH.

## Conclusion

In conclusion, we identified a network of reduced FC associated with PDP. The network included regions from key attentional and default mode networks and its association with hallucination severity, as well as with baseline and future motor symptoms, cognition, and attention underscores its relevance in PDP. Overall, these results highlight the importance of resting-state fMRI network analysis in uncovering neural differences related to hallucinations, and a deeper understanding of early-stage brain network dysfunction in PDP could help target therapeutic interventions towards the most effective neural systems.

## Supplementary Material

fcaf185_Supplementary_Data

## Data Availability

All analyses used publicly available packages and code, available at: https://github.com/marcellamontagnese/PDPrestingfMRI. No new data were generated. PPMI data, downloaded on 05 October 2021, can be accessed at: www.ppmi-info.org, and ICICLE-PD data are available by contacting the study lead at Newcastle https://www.bam-ncl.co.uk/iciclepd.
